# Which factors affect health care use among older Germans? Results of the German ageing survey

**DOI:** 10.1186/s12913-017-1982-0

**Published:** 2017-01-13

**Authors:** André Hajek, Jens-Oliver Bock, Hans-Helmut König

**Affiliations:** Department of Health Economics and Health Services Research, Hamburg Center for Health Economics, University Medical Center Hamburg-Eppendorf, Hamburg, Germany

**Keywords:** Health care use, Health care utilization, Andersen’s behavioral model, Longitudinal study, Older adults

## Abstract

**Background:**

It remains an open question how *changes* in predisposing, enabling, and need factors affect health care use. Consequently, we aimed to investigate how changes in these variables affect health care use in community-dwelling older persons longitudinally.

**Methods:**

Data from two waves of the German Ageing Survey (DEAS), a representative sample of the community-dwelling German population aged ≥40 years, was used. Predictors of visits to general practitioners and specialists as well as hospital stays during a 12-month period were analyzed by fixed effects regressions.

**Results:**

Regressions revealed that the need factors ‘self-rated health’ and the number of chronic diseases affected all measures of health care use (except for the number of chronic diseases on hospital care). An increased duration of physical activities increased GP visits. A decrease of excess weight decreased the number of specialist visits.

**Conclusions:**

Our findings underline the importance of need factors for health care use. Virtually none of the predisposing factors nor enabling resources affected health care use. These findings might indicate that individuals in the second half of life use health care services adequately, i.e. when medically indicated.

## Background

Health care use has long been an important issue in health policy. Defined as the meeting of supply and demand of health care, health care use includes in particular, hospital stays and physician visits, besides many other goods and services, such as pharmaceuticals or physiotherapy. Providing health care resources requires substantial financial efforts, and knowledge about determinants of health care use is important for health policy in order to manage health care use.

Andersen and Newman [1] developed a theoretical framework for analyzing determinants of health care use in 1973. Over the years, this behavioral model has been further developed to a version published in 1995 [2], which distinguishes three categories of *determinants of health care use*: *predisposing*, *enabling*, and *need factors*. The Andersen model is a widely used framework to examine determinants of health care use [3]. Therefore, this conceptual framework was used to select independent variables such as age or employment status.

*Predisposing factors* include, for example, demographic variables, like age and gender or the social structure. Social structure refers to the manner in which, a person can cope with problems or possess adequate means to solve problems. *Enabling resources* are financial and organizational factors that enable persons to use health care services. They are a prerequisite for the use of health care services [1]. The Andersen behavioral model distinguishes between personal and family resources as well as community-related resources. *Need factors* represent perceived or evaluated need for care, i.e. they either are based on the individual view upon his or her own health, or are assessed by a professional or by means of objective measures.

The differentiation into the three components of possible determinants in Andersen’s behavioral model is – beyond scientific reasons – also policy-oriented. Predisposing and enabling factors may influence health care seeking and might hint at inequity in access to health care. Thus, the goal of achieving equitable access to health care means that the influence of predisposing and enabling factors ought to be reduced, with need factors remaining the main reason for using health care services.

Many studies have analyzed health care use based on Andersen’s behavioral model [3]. It is well known that the actual determinants found in empirical studies are not always related to need factors. For example, for the German health care context, it has been recognized that women tend to use services in the outpatient sector more frequently than men do [4]. Health care use that is not related to need factors, however, might point to underuse, overuse or misuse of these services [5]. Therefore, it is essential to identify predisposing and enabling factors affecting health care use.

For the German health care context, some studies have thoroughly described the use of health care services [6, 7]. Germany’s health care system is characterized by a health insurance system that provides comprehensive protection against health care expenses. About 90% of the population are insured by social statutory health insurance (SHI) funds, while the remaining 10% are covered by private health insurances (PHI). For most members of SHI, the membership is compulsory, which is in particular, the case for employees below a certain income-threshold. Self-employed and employees above the income-threshold may opt for PHI. Contributions to SHI are income-related and independent of health status, whereas the contrary is the case for PHI. Both types of health insurance cover most expenses of inpatient and outpatient treatment, as well as for pharmaceuticals. Patients are guaranteed access to General Practitioners (GP), as well as specialists without further requirements, while hospital care can be used when being referred to by an outpatient physician or in case of an emergency. Patients of a SHI had to pay a small co-payment (€10 per quarter) until 2013 when using outpatient physician services, and €10 per day in a hospital. The waiting time for appointments with outpatient physicians is usually short [8]. As it is the case with other developed nations, in Germany, most visits to outpatient physicians and hospital stays are caused by the older population [9]. Accordingly, the older population accounts for a large proportion of health care costs, and the number of older people is likely to increase substantially in the next decades.

Beyond describing health care use in Germany, patterns of health care use have been analyzed comprehensively by various studies [4, 10–12]. Yet, these studies were limited to cross-sectional designs. Consequently, these studies gave important insights into associations between, certain predisposing as well as enabling factors and health care use. However, causal mechanisms between them still need to be investigated. This is important to derive interventional strategies.

There are only a few longitudinal studies investigating the predictors of health care use [13–15]. However, these longitudinal studies mainly used a *static* set of baseline predictors to predict subsequent health care use. Thus, it remains an open question how *changes* in independent variables affect health care use. Consequently, the aim of the longitudinal study was to investigate how changes in predisposing, enabling, and need factors affect health care use in a representative sample of community-dwelling older persons. This might help to gain insights into the causal relationship between the independent variables and health care use. Knowing the predictors of health care use is important for health policy. It is a main goal of the German health care system to provide universal access to health care and reduce barriers to access. If predisposing factors and enabling resources, rather than need factors were related to health care use, this would point to inadequacy of health care use and inequity in access to health care, which policy makers would have to address. For the target group of individuals in the second half of life aged 40 years and above, little is known about determinants of health care use. However, due to demographic shifts, the number of individuals in old age is likely to increase substantially, emphasizing the importance of this study and the need to focus on elderly adults. Thus, this study might be important for policy-makers to identify approaches to modify health care behavior.

## Methods

### Sample

Data came from the public release of the German Ageing Survey (DEAS). This is a population-based, representative (national probability sampling) survey of the community-dwelling population aged 40 years and older in Germany. Data was provided by the Research Data Centre of the German Centre of Gerontology (DZA). Baseline recruitment took place in 1996; 10,608 randomly selected inhabitants of Germany above the age of 40 years were asked via mail to participate in the study. Addresses had been taken from registry offices who own data of all inhabitants due to compulsory registration. In the waves 3 and 4, individuals who consented to participate were interviewed face-to-face by trained staff via computer-assisted personal interviewing (CAPI). These interviews covered, for example, general socio-demographic information and many general topics of aging. Following the interview, participants were asked to answer a standardized questionnaire that included more personal topics like psychological factors (e.g., satisfaction with life or self-efficacy) or illnesses. The data used in this study were derived from the subsamples who sent back the questionnaire.

We restricted our analysis to the waves 3 (2008) and 4 (2011), since physical activities were comprehensively assessed only from wave 3 onwards. 8200 individuals participated in the third wave, whereas 4855 individuals participated in the fourth wave. The response rate for the third wave was 38%, and it was 56% in the fourth wave. The survey in 2008 considered a cross-sectional sample as well as panel sample of study participants, whereas the most recent survey in 2011 considered only panel participants. Thus, the sample sizes differed considerably between those two waves. 6205 community-dwelling individuals were interviewed for the first time in the third wave, whereas 1995 individuals had already been interviewed in former waves. For example, reasons for not participating in the fourth wave were that 23% did not participate anymore, 10% could not be contacted, 5% for reasons of health, and 3% had died or moved to another country. More details for the third wave have been reported elsewhere [16].

Individuals were only included in our regression analysis if they had *changes* in the outcome variables between the third and fourth wave, resulting in 1372 individuals (with specialist visits as outcome variable). Please refer to the chapter “Statistical analysis” for more details regarding regression analysis. Further details regarding the sampling frame and the sample composition can also be found elsewhere [17].

Research carried out on humans in the German Ageing Survey were in compliance with the Helsinki declaration.

### Outcome: health care use

Assessed health care use included outpatient physician services and hospital treatment in the past 12 months, measured as:

- Number of visits to GP, including home visits
- Number of visits to specialists, including home visits: Internists, gynecologists, ophthalmologists, orthopedists, ear, nose, and throat specialists, neurologists, psychiatrists, dermatologists, urologists, and other specialists (open answer)
- Number of days in hospital

Individuals reported the frequency of visits to GPs and specialists (“never”, “once”, “2–3 times”, “4–6 times”, “7–12 times”, “more often” (open answer). They were recoded as follows: never = 0; once = 1; 2–3 = 2.5; 4–6 times = 5, 7–12 times = 9.5; more often = 13. The hospital stays were dichotomized (0 = no hospital stay, 1 = hospital stay).

### Independent variables

*Predisposing factors* were included as follows: Age, gender, place of birth (whether Germany or abroad), family status (married, living together with spouse, others (married, living separated from spouse; divorced; widowed; never married)), employment status (working; retired; not employed) and educational level (International Standard Classification of Education (ISCED) [18]). The ISCED contains the following categories: low (ISCED 0–2: respondents without formal vocational qualification), medium (ISCED 3–4: respondents with vocational training at work or at school, including respondents with a higher general school certificate without professional training) and high (ISCED 5–6: respondents with completed professional development training (professional, master or technical school, university of cooperative education or academies) and respondents with completed university studies (university or university of applied science).

*Enabling resources* were included as self-rated accessibility of doctors and (log) monthly equivalence income (new Organisation for Economic Co-operation and Development (OECD) equivalence scale) in Euro. The self-rated accessibility was quantified based on the item “There are not enough doctors and pharmacies in the vicinity” (no; yes).

*Need factors* covered morbidity and subjective health. Morbidity was quantified by using the total number of chronic diseases (adapted from the Charlson Comorbidity Index [19]) such as cancer, depression, diabetes or osteoporosis. Subjective health was included based on a self-rating scale: 1 = “very good” to 5 = “very bad”. Additionally, lifestyle factors represented need factors, operationalized as physical activity, excess weight and current smoking status (yes; no). Physical activity was quantified by summing the total approximated time per week (in hours and minutes) for (i) endurance sports (e.g., swimming, long-distance running, jogging, cycling), (ii) team sports or games (e.g., handball, soccer, tennis, volleyball, basketball, squash, badminton), and (iii) strength training or combat sports (e.g., weightlifting, bodybuilding, karate, judo, including activities in a gym) [20]. Excess weight was self-reported and defined according to the World Health Organization (WHO) thresholds for Body Mass Index (BMI): underweight (BMI < 18.5 kg/m^2^), normal weight (18.5 kg/m^2^ ≤ BMI < 25 kg/m^2^), overweight (25 kg/m^2^ ≤ BMI < 30 kg/m^2^), and obesity (BMI ≥ 30 kg/m^2^).

### Statistical analysis

Fixed effects (FE) regressions were used to estimate the effect of time-dependent regressors on health care use. When time-constant unobserved factors, such as genetic disposition, are systematically correlated with the predictors, random effects (RE) regressions lead to inconsistent estimates [21]. Contrarily, FE regressions provide consistent estimates even when time-constant unobserved factors are correlated with the predictors. Consequently, in our case, FE regressions are the method of choice (indicated by Hausman tests).

FE regressions only use intraindividual changes (within-variation). This means that changes within individuals over time were examined. For this reason, the FE estimator is also called ‘within-estimator’. Consequently, solely time-dependent variables (such as income or self-rated health) can be included as predictors in FE regressions. Thus, time-constant variables, such as gender or education, cannot be included as independent variables in FE regressions. However, time-constant can be included in the model as moderator variables. Consequently, in additional models, it was tested whether need factors – proven to be significant in main regression models – were moderated by the predisposing factors gender or education, respectively.

While the predictors of hospital stays (binary) were estimated by using conditional FE logistic regressions, the predictors of GP and specialist visits (count data) were estimated by using FE Poisson regressions. The level of significance was set at α = .05. Statistical analysis was conducted using Stata Release 14 (Stata Corp., College Station, Texas; http://www.stata.com/new-in-stata).

## Results

### Sample characteristics

The pooled (wave 3 and wave 4) median for GP visits was 2.5 and the median for specialist visits was 2. 90% of participants had at least one visit to a GP during the 12 months preceding the interview and about 65% used services provided by a specialist. Moreover, about 20% were hospitalized in the last 12 months.

Since we were interested in the intra-individual changes, individuals were only included in the sample if they had changes in the outcome variable between wave 3 and wave 4. Descriptive statistics for individuals included in FE regression analysis with GP visits as outcome variable are depicted in Table 1. As for the time-constant variables (not included in FE regressions as independent variables), the majority was female (52.2%) and had a medium educational level (50.8%). In addition, 86.9% of the individuals were born in Germany.

**Table 1:** Sample Characteristics for Individuals included in fixed effects regressions (with GP visits as outcome variable, waves 3–4, pooled)

Time-constant variables (not included as independent variables in FE regressions)	Female: N (%)	1372 (52.2)
	Low education: N (%)	173 (7.9)
	Medium education: N (%)	1112 (50.8)
	High education: N (%)	906 (41.3)
	Place of birth: Germany: N (%)	1902 (86.9)
Predisposing factors	Age (in years): Mean (SD)	64.3 (11.2)
	Married, living together with spouse: N (%)	1952 (74.2)
	Working: N (%)	860 (32.7)
	Retired: N (%)	1498 (57.0)
	Other: not employed: N (%)	272 (10.3)
Enabling resources	Equivalence income: Mean (SD)	1794.8 (1782.4)
	Self-rated accessibility of doctors = Yes (accessible): N (%)	2112 (80.3)
Need factors	Self-rated health (from “very good” to “very bad”): Mean (SD)	2.4 (0.8)
	Number of chronic diseases: Mean (SD)	2.4 (1.8)
	Underweight: N (%)	20 (0.8)
	Normal weight: N (%)	1029 (39.1)
	Overweight: N (%)	1069 (40.6)
	Obesity: N (%)	512 (19.5)
	Currently smoking: N (%)	366 (13.9%)
	Physical activities (in hours per week): Mean (SD)	2.9 (3.6)
	Observations	2630

The number in the variables education and place of birth do not sum up to 2630 due to missing values

Mean age was 64.3 years (±11.2 years), ranging from 40 to 95 years. The majority were married, living together with their spouse (74.2%), retired (57.0%) and had a mean net income of €1,794.8 (±€1,782.4). 80.3% were satisfied with the accessibility of doctors. The mean self-rated health was 2.4 (±0.8) and the mean number of chronic diseases was 2.4 (±1.8). Furthermore, most of the individuals either had a normal weight (39.1%) or were overweight (40.6%). Only 13.9% were current smokers. The mean duration of physical activities (in hours per week) was 2.9 (±3.6).

The descriptive statistics for individuals included in FE regression analysis with specialist visits as outcome variable was almost the same. However, descriptive statistics for individuals included in FE regression analysis with hospital stay as outcome variable were somewhat different. For example, individuals had a mean self-rated health of 2.7 (±0.9) and a mean number of chronic diseases of 3.1 (±2.0). The median for GP visits was 2.5 and the median for specialist visits was 4.0.

### Main regression analysis

Table 2 presents the results of FE-regressions for GP visits and specialist visits. GP visits increased with changes in employment status (predisposing factor) from ‘working’ to ‘retired’ or ‘not employed’. Other predisposing factors as well as enabling resources did not affect GP visits significantly. GP visits increased with decreased self-rated health and the number of chronic diseases (both need factors). Furthermore, while GP visits increased with an increased duration of physical activities, the other lifestyle factors (BMI categories and the current smoking status) did not affect this outcome variable significantly.

**Table 2:** Predictors of GP and Specialist visits. Results of fixed effects poisson regressions (Waves 3–4)

	Independent variables	(1)	(2)
		GP	Specialist
Predisposing factors	Age (in years)	0.00576	−0.00776
		(0.00691)	(0.00886)
	Other marital statuses (Ref.: Married, living together with spouse)	−0.146	−0.0811
		(0.0960)	(0.106)
	Retired (Ref.: Working)	0.165*	−0.0138
		(0.0801)	(0.126)
	Other: not employed	0.181*	0.0822
		(0.0759)	(0.101)
Enabling resources	(Log) equivalence income	0.0754	0.0993
		(0.0544)	(0.0718)
	Self-rated accessibility of doctors (Ref.: No accessibility of doctors)	0.00891	0.0131
		(0.0375)	(0.0539)
Need factors	Self-rated health (from “very good” to “very bad”)	0.108***	0.200***
		(0.0268)	(0.0338)
	Number of chronic diseases	0.0425**	0.0557**
		(0.0140)	(0.0174)
	Underweight (Ref.: Normal weight)	−0.147	0.0770
		(0.177)	(0.217)
	Overweight	−0.0217	−0.156*
		(0.0570)	(0.0726)
	Obesity	−0.00928	−0.238*
		(0.0778)	(0.113)
	Currently smoking (Ref.: Currently not smoking)	0.0463	−0.188
		(0.111)	(0.163)
	Physical activities (in hours per week)	0.0204**	0.0161^+^
		(0.00739)	(0.00867)
	Observations	2630	2744
	Number of Individuals	1315	1372

Beta-Coefficients were reported; Cluster-robust standard errors in parentheses; *** *p* < 0.001, ** *p* < 0.01, * *p* < 0.05, ^+^ *p* < 0.10

Specialist visits also increased with decreased self-rated health and the number of chronic diseases. Additionally, specialist visits increased with changes from ‘normal weight’ to excess weight. None of the predisposing factors nor the enabling resources affected specialist visits significantly.

Table 3 depicts the results of FE-regressions for hospital stay. The probability of hospitalization significantly increased with higher age, changes in the employment status from ‘working’ to’not employed’, and decreased self-rated health. Other predictors did not reach statistical significance.

**Table 3:** Predictors of hospital stay. Results of conditional fixed effects logistic regressions (Waves 3–4)

	Independent variables	Hospital stay (Ref.: No)
Predisposing factors	Age (in years)	0.909*
		(0.842–0.982)
	Other marital statuses (Ref.: Married, living together with spouse)	0.989
		(0.315–3.099)
	Retired (Ref.: Working)	0.739
		(0.284–1.928)
	Other: not employed	2.372*
		(1.013–5.556)
Enabling resources	(Log) equivalence income	0.751
		(0.407–1.385)
	Self-rated accessibility of doctors (Ref.: No accessibility of doctors)	1.042
		(0.629–1.726)
Need factors	Self-rated health (from “very good” to “very bad”)	1.770***
		(1.349–2.322)
	Number of chronic diseases	1.084
		(0.942–1.248)
	Underweight (Ref.: Normal weight)	0.509
		(0.0484–5.350)
	Overweight	0.929
		(0.484–1.786)
	Obesity	0.878
		(0.337–2.284)
	Currently smoking (Ref.: Currently not smoking)	0.534
		(0.169–1.687)
	Physical activities (in hours per week)	1.042
		(0.966–1.124)
	Observations	770
	Number of Individuals	385
	Pseudo R^2^	0.08

Odd ratios reported; 95% CI in parentheses; *** *p* < 0.001, ** *p* < 0.01, * *p* < 0.05, + *p* < 0.10

### Additional models

In additional models (not shown), it was tested whether the effect of need factors found to be significant were moderated by education and sex (i.e. education x self-rated health, education x number of chronic diseases; sex x self-rated health; sex x number of chronic diseases). However, the interaction terms did not reach statistical significance.

## Discussion

### Main findings

The need factors ‘self-rated health’ and the number of chronic diseases affected all considered measures of health care use, except for the number of chronic diseases on hospital care. This effect did not vary by gender nor educational level. This might indicate that inequalities in the access to health care services did not exist. An increased duration of physical activities increased GP visits. Changes to excess weight decreased the number of specialist visits. Generally, age is positively associated with weight in older adults [22]. A possible explanation for our findings might be that losing weight in older age is often associated with adverse health outcomes [23], such as functional or cognitive decline, which might be associated with specialist visits (e.g., neurologist). Furthermore, there was no robust effect of predisposing factors and enabling resources on any measure health care use.

### Need factors

In our study, mainly need factors affected health care use. Need factors were operationalized as self-rated health, number of chronic conditions, and lifestyle factors. ‘Self-rated or perceived health’, which has often been identified as an important need factor [3], affected health care use with worse subjective health leading to more health care use in our study. This association has also been reported in previous cross-sectional studies [24–27]. Findings from longitudinal data sets substantiate this finding [13–15]. This means that longitudinal studies in addition to our study provide first insights into the causal relationship between objective health and health care use. This indicates that objective health is likely to be a causal factor of health care use.

In addition, objective health measures have also been used frequently to represent need factors in the Andersen behavioral model [3]. Our results show a statically significant effect of an increasing number of chronic conditions on higher health care use in outpatient care, i.e. GP and specialist visits. Thus, the present study substantiates the knowledge about the often-found (cross-sectional) association of worse objective health and health care use [4, 25–29].

There are numerous longitudinal studies focusing on the impact of *specific illnesses* on health care use. For instance, Parkinson’s disease [30], overactive bladder [31], comorbid diabetes and depression [32], and inflammatory bowel disease [33] were found to increase health care use. In addition to these studies, the present study examined the impact of *total number of chronic conditions* (morbidity) on health care use. In sum, our results indicate that there might be causal relationship between health-related need factors, both perceived and objectively measured, and health care use. It is worth emphasizing that the effect of these need factors did not vary by gender and educational level.

It appears highly plausible that changes in these need factors are strongly associated with health care use. Thus, the occurrence of need factors implies that certain symptoms or signs of an illness were observed by the patient. Such a need for help usually directly entails visits to physicians or hospitals, because most outpatient services are virtually free of charge and waiting times are low in the German health care system, so that patients can have their symptoms checked immediately by professionals.

Apart from health-related need factors, we considered lifestyle factors as need factors and found physical activities to increase GP visits. Apparently, this finding appears to be counterintuitive, as one could assume that, in general, physical activities are associated with a healthier lifestyle, which should be associated with fewer visit to physicians. For example, Thode et al. [4] did not find a statistically significant association between physical activity and number of physician visits. Yet in our longitudinal study, changes towards more physical activities entailed more GP visits. It might be that more physical activities lead to more GP visits due to injuries resulting from sports. Besides, changes in smoking behavior did not affect the outcome variables and excess weight affected specialist visits only, indicating that changes in health care use are predominantly driven by health-related need, rather than lifestyle factors.

### Enabling resources and predisposing characteristics

We found enabling resources (income and perceived access to primary health care services) not to be predictive for health care use. Findings about associations of income and health care use are ambiguous. There are international studies reporting associations in either direction [25, 34], while others found no association [28]. Cross-sectional evidence from the German health care system based on routine data showed higher income to be associated with fewer physician contacts [11]. No association of income and costs in any health care sector (based on use) was found for older adults in Germany based on survey data [10]. For the German health care system, this finding appears not surprising, since the vast majority of health care goods and services are free of charge and existing co-payments are rather low. For outpatient physician treatment, patients insured by SHI in the years 2004 to 2012 had to pay only a small co-payment of €10 per first contact to a doctor in a quarter while any further contact in the quarter was free of charge.

Other studies used socioeconomic status (SES) instead of income. For example, an earlier survey-based study did not find an association of SES and total outpatient physician contacts in Germany [4]. In contrast, recent research found high SES to be associated with total direct costs [6], mainly driven by outpatient physician and hospital treatment. Both studies included SES instead of income, so that they cannot be compared directly with our results. In total, the relation between SES/income and health care use depends on the concrete context, such as the health care system or study population.

In our study, changes in perceived accessibility did not affect health care use. Recent cross-sectional analyses showed an association between perceived access and health care use in Taiwan [35]. For Germany, objective accessibility, measured as ratio of number of physicians and population, was not associated with GP visits nor specialist visits [4]. There is a controversy for Germany whether there is an objective shortage of GPs (in particular in certain rural areas) [36]. Our study indicates that there were at least perceived difficulties to access health care services, yet without affecting health care use.

Among the predisposing factors, only retirement or leaving one’s job for other reasons increased GP visits. One reason for this might be a change in health behavior due to job loss [37, 38]. No other predisposing factors affected health care use in each sector. This indicates that there is no consistent causal influence of predisposing factors on use of outpatient or hospital care, which suggests that individuals use health care services appropriately, i.e. when medically induced. This makes sense, considering the health care structure in Germany that provides free access to health care services and comprehensive coverage of health care expenses by health insurances. Our data also suggest that there are no social inequalities in health care use (outpatient physician services and hospital treatment) in Germany. This is in contrast to other countries such as England [39] or the United States [40], where social inequalities in health care use exist. Changes in marital status were not associated with the outcome variables. A possible explanation is that changes in marital status might result in negative health effects like depressive symptoms in cases of divorce or widowhood. However, these factors are mainly captured by the need factors included in our regression model.

### Strengths and limitations

This is the first *longitudinal* study investigating the predictors of health care use in older adults in Germany. By using FE regressions, time-constant unobserved heterogeneity was taken into consideration, providing consistent estimates. Moreover, it is worth emphasizing that we draw on a large representative sample of community-dwelling older persons in Germany. Additionally, unlike previous studies, we were able to assess physical activity accurately (albeit self-assessed with the corresponding potential drawback regarding the validity).

However, one limitation is that our estimates might be biased downwards for reasons of panel attrition in the German Ageing Survey [41]. Even though main lifestyle factors were included in regression analysis, the effect of changes in alcohol consumption could not be investigated for reasons of data availability. Also due to reasons of data availability, we could not differentiate between members of private and statutory health insurance. However, in Germany, the SHI covers about 90% of the population. Thus, the influence of PHI is rather small. Another limitation is that self-rated health was measured using one item. Thus, mono-operationalization-bias might be a threat to the validity of our results. Furthermore, the self-rated BMI was used. Consequently, it is likely that the BMI is biased downwards.

## Conclusions

While our study investigated the quantitative *use of health care*, further research is required regarding the *access* and *quality of care*. Furthermore, our study investigated *outpatient physician and hospital treatment*, whereas further research is required regarding the *prevention* and *health promotion*.

Our findings underline the importance of need factors for health care use in Germany. This effect did not vary by gender nor educational level. Besides, our analyses show that it is important to take into account time-constant unobserved factors. Virtually none of the predisposing factors nor enabling resources affect health care use. This might indicate that individuals use health care services adequately, i.e. when medically indicated, stressing the meaning of need factors for the German health care system.

## Abbreviations

BMI: Body mass index
DEAS: German ageing survey
DZA: German centre of gerontology
FE: Fixed effects
GP: General practitioner
ISCED: International standard classification of education
OECD: Organisation for economic co-operation and development
PHI: Private health insurance
RE: Random effects
SHI: Statutory health insurance
WHO: World Health Organization
